# Personality disorders and psychosocial problems in a group of participants to therapeutic processes for people with severe social disabilities

**DOI:** 10.1186/1471-244X-11-192

**Published:** 2011-12-11

**Authors:** Carlos Salavera, José M Tricás, Orosia Lucha

**Affiliations:** 1 Departament de Psychology and Sociology. Education Faculty, Zaragoza University, C/San Juan Bosco, 7. 50009 Zaragoza, Spain; 2 Physiotherapy Research Unit, Zaragoza University, C/Domingo Miral s/n, 50009 Zaragoza, Spain

## Abstract

**Background:**

Homeless people have high dropout rates when they participate in therapeutic processes. The causes of this failure are not always known. This study investigates whether dropping-out is mediated by personality disorders or whether psychosocial problems are more important.

**Method:**

Eighty-nine homeless people in a socio-laboral integration process were assessed. An initial interview was used, and the MCMI II questionnaire was applied to investigate the presence of psychosocial disorders (DSM-IV-TR axis IV). This was designed as an *ex post-facto *prospective study.

**Results:**

Personality disorders were very frequent among the homeless people examined. Moreover, the high index of psychosocial problems (axis IV) in this population supported the proposal that axis IV disorders are influential in failure to complete therapy.

**Conclusion:**

The outcomes of the study show that the homeless people examined presented with more psychopathological symptoms, in both axis II and axis IV, than the general population. This supports the need to take into account the comorbidity between these two types of disorder among homeless people, in treatment and in the development of specific intervention programs. In conclusion, the need for more psychosocial treatments addressing the individual problems of homeless people is supported.

## Background

Homeless people live in city streets and temporary shelters because of successive sudden and traumatic rupture of family, social and labor ties [1]. They form one of the most vulnerable and disadvantaged groups in society [2]. Their situation is also related to a low quality of life and to a high rate of physical and psychic diseases [3]. They suffer obvious mental deterioration because they inhabit the street [4-6] and they are considered to represent the maximum level of social exclusion in modern society [7].

Personality disorders (PD) are the mental pathologies most obviously present in this population. Sometimes several disorders coexist [8]. It also must be considered that such psychosocial conditions existed prior to the individuals' departure from their homes [9].

There has been extensive work on how to deal with PD [10-14]. Recently, there has also been much research on PD in populations being treated for addictions [15-17]. Treatments for psychosocial disorders remain less fully developed because of the possible variables in community treatment [18].

In this study, our aim was to investigate which factors (psychosocial or personality disorders) are more important contributors to treatment dropout among homeless people.

## Methods

### Participants

The sample consisted of homeless people (N = 89) who registered at the center for the homeless under consideration. The 89 subjects in the study were selected from among 96 people who underwent an inclusion assessment for homeless people based on the following criteria; (a) being homeless; (b) more than two months' stay in the center; (c) voluntary participation in the study; and (d) a long enough stay to complete the study.

### Assessment measurements

- Initial assessment interview: Initially, a structured individual interview was conducted in order to make a diagnosis. In this interview the most significant data were collected: age, marital status, educational level, age and cause of onset of transient life, familial relationships, alcohol and drug consumption and previous psychological treatment.

- Millon Multiaxial Clinical Inventory MCMI-II [19]: The MCMI-II provides theoretically derived measures which predict diagnostic probabilities and a wide range of cognitive, affective, perceptual, interpersonal, and behavioral clinical attributes (Millon, 1987). The questionnaire comprises 175 questions, with true or false choices, which are answered in 25-30 minutes.

- Axis IV disorder evaluation interview of DSM-IV-TR: A structured individual interview was carried out in order to diagnose the problems in relation to axis IV psychosocial disorders. In this interview, data about different factors of the problem were collected: problems with the family of origin or the created family, problems at school, work, home, economic, health or legal problems.

### Procedure

The study was conducted through interviews carried out by clinical psychologists; they performed the Initial Assessment interview. In order to measure the disorders in axis II of DSM-IV-TR, after analyzing different personality assessment tools, the MCMI II Millon Multiaxial Clinical Inventory was selected because it is quick and easy to apply and provides an opportunity to obtain more relevant information about the subjects. Finally, to assess problems in axis IV of DSM-IV-TR (psychosocial disorders), a complementary diagnostic interview was carried out, in which the problems relating to that axis were analyzed (family of origin, created family, school, work, home, economic, health and legal).

All the subjects were males, over 18 and with sufficiently long pathways in the treatment process for the data to be collected and an objective assessment made. They also signed an informed consent form for participation in the research.

Both the interviews and the MCMI II were applied and corrected by the two clinical psychologists. The study considered the presence of a personality disorder when the score in the rate-base (RB) of the MCMI II was more than 74. Also, in order to measure adherence to treatment and achieve honesty and reliability in the interviews and tests, all cases were examined after two months' stay in the center.

For statistical analysis of the data, the statistical program SPSS 15.0 version was used. A descriptive analysis was performed (maxima, minima, averages and standard deviation) for each of the variables. In all cases the significance level used was 5%. Crossed variables and bilaterally correlated analysis were also carried out. The study conformed to the policies established by the Code of Ethics of the World Medical Association (Declaration of Helsinki) and ethics approval was obtained from the Doctoral Commission of the University of Zaragoza.

## Results

In this section (table 1), the large number of young homeless people in the integration process stands out; nearly one in every four subjects was younger than 30. The percentage of persons older than 50 years was surprisingly low (7.9%). This could indicate the low interest of people at this age in integration processes, an obvious result of the damage suffered during their stay in the street and their disillusionment with previous processes (Cabrera, 1998; Salavera *et al*, 2009). Regarding their marital status, although 60.7% (N = 47) were single parents, with or without maintenance obligations, the low number of people with a partner during their stay at the center (3.3%) stands out, and so does the high number of breakups among people who have had a partner (36%). But above all, the number of people who had not established a stable relationship with their partner stands out. These data are consistent with other studies [20]. From the research done, a low level of schooling can be seen, mainly because of early incorporation into the labor market.

**Table 1:** Sociodemographic features (N = 89)

AGE	N	%
Average	37,86	
Range	(22-52)	
< 30 years	19	(21,3%)
30-39 years	32	(36%)
40-49 years	31	(34,8%)
> 50 years	7	(7,9%)
**MARITAL STATUS**		
		
Sigles	54	(60,7%)
Separated/Divorced	32	(36%)
De facto partnership	3	(3,3%)
**SCHOOLING**		
		
School Certificate	31	(35,2%)
EGB	42	(47,2%)
FP	10	(11,2%)
BUP	5	(5,6%)

**Figure 1:**
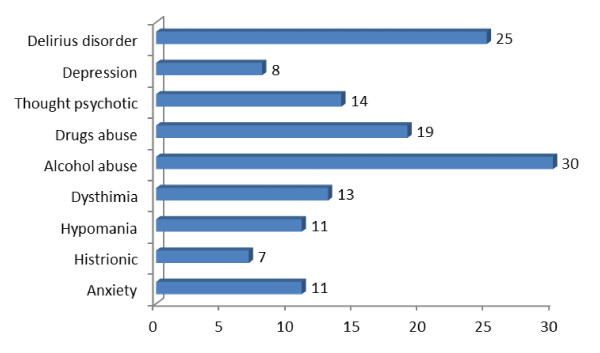
**Organic disorders in the population**.

Moreover, organic problems in the subjects were analyzed (Figure 1), alcohol and drugs abuse high incidence were observed, as well as a low frequency of cognitive damage.

### Axis II variables

Table 2 shows the Millon Multiaxial Clinical Inventory MCMI-II results for the current study. The antisocial (35.1%, N = 27), dependent (29.9%, N = 23), compulsive (28.6%, N = 22) and narcissistic (28.6%, N = 22) disorders show higher scores, RB > 74. It is remarkable that some subjects gave test results showing they could have one or more subscales with high scores.

**Table 2:** MCMI II Scores (N = 89)

	Minimum	Maximum	Average	***S.D***.	F	**Signif**.	% RB > 74
Schizoid	0	117	59,98	26,543	3,925	,051	24 (27%)
Phobic	2	103	50,82	29,466	1,772	,187	22 (24,7%)
Dependent	0	108	55,93	29,523	6,670	,011	25 (28,1%)
Histrionic	5	100	51,66	24,518	1,039	,311	18 (20,2%)
Narcissist	0	109	56,61	24,982	,608	,438	24 (27%)
Antisocial	0	121	65,84	28,324	2,893	,093	34 (38,2%)
Aggressive	0	120	54,64	28,161	,828	,365	19 (21,3%)
Compulsive	5	120	65,82	26,168	1,268	,263	29 (32,6%)
Passive	0	103	41,93	27,971	1,632	,205	13 (14,6%)
Self-destructive	0	109	52,28	25,259	,090	,765	12 (13,5%)
Schizotipical	5	117	55,70	25,988	2,112	,150	18 (20,2%)
Limit	0	112	45,48	27,667	1,462	,230	11 (12,4%)
Paranoid	8	118	62,43	22,869	,061	,805	19 (21,3%)

**Figure 2:**
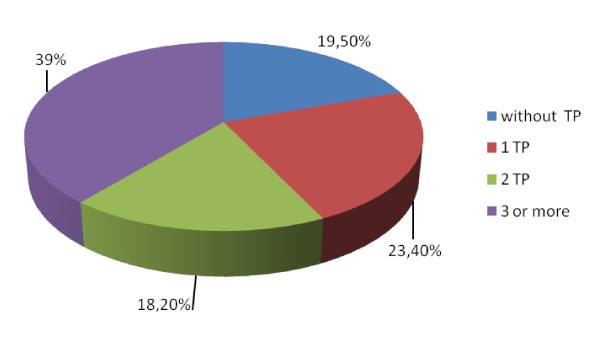
**Personality disorders in the population**.

The number of personality disorders was also analyzed in every subject (Figure 2). Some subjects in homeless integration treatment had no personality disorder (19.5% of cases, N = 15), but some presented one (23.4%, N = 18), two (18.2%, N = 14) or three or more (39%, N = 30) personality disorders.

### Axis IV Variables

Each participant was interviewed in order to diagnose the various disorders in axis IV (DSM-IV-TR). This was evaluated separately by both clinical psychologists to ensure inter-tester reliability (table 3).

**Table 3:** Psychosocial disorders (axis IV) present in the subjects studied.

	Present problems	F	**Sig**.
Origin family problems	73 (82%)	5,623	,020
Created family problems	38 (42,7%)	,229	,633
Shool problems	31 (35,2%)	,420	,519
Work problems	36 (40,4%)	16,643	,000
Home problems	89 (100%)	.	.
Economical problems	89 (100%)	.	.
Health problems	33 (37,1%)	1,569	,214
Arrested	48 (53,9%)	,275	,601

Moreover the importance both axes (II and IV) in the rate of dropping out from treatment processes was analyzed. Among the participants in the study, 33 (37.08%) failed to complete and 56 (62.92%) completed the process. In table 4, the disorders of the subjects are indicated, and also the percentages of total dropouts and integrations.

**Table 4:** Dropouts according to axis II and IV (N = 33)

	Disorders seen in dropouts	Dropouts in relation to the total simple size
Schizoid	12 (36,36%)	12 (13,48%)
Phobic	10 (30,30%)	10 (11,23%)
Dependent	4 (12,12%)	4 (4,49%)
Histrionic	8 (24,24%)	8 (8,98%)
Narcissist	11 (33,33%)	11 (12,35%)
Antisocial	16 (48,48%)	16 (17,97%)
Aggressive	7 (21,21%)	7 (7,86%)
Compulsive	10 (30,30%)	10 (11,23%)
Passive	7 (21,21%)	7 (7,86%)
Self-destructive	6 (18,18%)	6 (6,74%)
Schizotipical	9 (10,11%)	9 (10,11%)
Limit	7 (21,21%)	7 (7,86%)
Paranoid	10 (30,30%)	10 (11,23%)

Origin family	10 (30,30%)	10 (11,23%)
Created family	20 (60,60%)	20 (11,23%)
School	13 (39,39%)	13 (14,60%)
Work	22 (66,66%)	22 (24,71%)
Home	33 (100%)	33 (100%)
Economical	33 (100%)	33 (100%)
Health	15 (45,45%)	15 (16,85%)
Arrested	19 (57,57%)	19 (21,34%)

Finally, correlations between organic, personality and psychosocial disorders were analyzed (table 5).

**Table 5:** Correlations between personality, organic and psychosocial disorders

		Origin family	Created family	School	Work	Home	Economic	Health	Arrested
Organic disorders	Anxiety								
	Hysteriform								
	Hypomania								-,238(*)
	Dysthymia					,614(**)	,300(**)		
	Alcohol abuse								-,260(*)
	Drugs abuse								-,383(**)
	Thought psychotic								
	Depression	,267(*)							
	Disorder delirious								

Personality disorders	Schizoid					,241(*)			
	Phobic					,261(*)			
	Dependent								
	Histrionic								
	Narcissistic								
	Antisocial								
	Aggressive								
	Compulsive	,236(*)							
	Passive								
	Self-destructive				,270(*)				
	Schizotypical				,212(*)		,212(*)		
	Limit								
	Paranoid								

The high prevalence of personality disorders (75.3%), the high number of personality disorder/person present in the sample (X = 2.84) and the homogeneity presented in the psychosocial problems in the population (remember that issues such as home environment, economy, employment status... are part of the definition of homelessness), makes difficult to attribute to personality disorders, valued in general, the presence of psychosocial disorders. Something similar happens in the case of organic disorders and their relation to psychosocial problems. In this case, the sample (homeless people for a long time), has an important organic impairment. Analyzing the correlations between personality disorders, with organic and disorders (table 5), we conclude that they are so linked in the sample that it is difficult to establish causal relationships between them other than those outlined in this table.

## Discussion and conclusions

This study focused on identifying the cause of dropping out from the integration process, evaluating whether the personality disorders or the psychosocial problems found in homeless people were more prominent.

According to the data, the typical profile of a homeless person would be one with more than one PD (*X *= 2.84), who usually presents with psychosocial problems, mostly home and economic problems in the sample studied, but also with serious problems in axis IV. The axis II disorders from which the homeless people suffered belonged mainly to the group of interpersonal and ambivalent conflicting problems. The disorders most commonly found were: antisocial: 38.2%; compulsive: 32.6%; dependent: 28.1%; narcissistic and schizoid: 27.0%, very similar percentages to those found in previous studies [15,21].

As expected, the most common disorders in the sample were related to interpersonal problems (dependent, narcissistic and antisocial), along with one belonging to the group of ambivalent personalities with conflicts (compulsive). This explains the correlation between these types of PD and the psychosocial problems as the main cause for dropping out. This, analyzed using Millon's typology [22], shows a person looking for independence not by his own self-confidence but by the distrust of others, with frequent failures in their obligations and with irresponsible and transgressive behavior (ASPD), expressively arrogant and interpersonally exploitative, ignoring coexistence rules (NPD), with serious internal divisions from which he cannot escape (CPD), avoiding adult responsibilities and self-assertiveness, and with a lack of functional competence (DPD)

The disorder most prevalent in the study population (one in every three subjects) was the antisocial personality disorder (ASPD); in the normal population it appears in only 3%, which rises to 75% among prisoners [23].

It is surprising that, along with ASPD, there was a high prevalence of the dependent personality (DPD). This was found in 29.9% of the subjects, three times the incidence obtained in previous studies. It is found in 3% of the clinical population and in 10% of the general population [14]. The fact that all the subjects were male makes the implication of this result even more far reaching. Subjects with DPD adhered well to treatment, with a low dropout rate.

In this study it was shown that 28.6% (N = 22) of the subjects can be considered to have a narcissistic personality disorder (NPD), more than reported in previous studies. This can be explained because in pathological narcissism, self-esteem is disturbed [24] and becomes fragile [25], something that occurs in homeless people. The prevalence of the narcissistic personality disorder in a general population has been reported to be 1% [26]. Other researchers have found a greater NPD rate [27], for instance 22% of the adult clinical population, and both are far from the results of our study.

As a preliminary conclusion, a high prevalence of PD was observed in this study, well above the rate found from epidemiological data concerning the general population [28].

Regarding the disorders in axis IV, every subject experienced home and economic difficulties, typical features of homeless people. Moreover, work problems, and difficulties with created family or the person's legal processes, have a marked impact on dropping-out, even greater than the personality disorders (axis II). This finding is consistent with studies concerning how the existence of a personality disorder influences the treatment dropout rate [29]. It can be said that the subjects in the sample with more than one type of personality disorder have a worse prognosis, and comorbidity between axes II and IV is a complicating factor.

The outcomes of the study show that the homeless people examined present with greater psychopathological symptoms, both in axis II and in axis IV, than the general population. This determines the rate of dropping-out from treatment processes. Our findings indicate the need to take the comorbidity between the two types of disorders in homeless people into account, both in treatment and in the development of specific intervention programs.

## List of abbreviations

ASPD: Antisocial Personality Disorder; CPD: Compulsive Personality Disorder; DPD: Dependent Personality Disorder; DSM-IV-TR: Diagnostical and Stadistical Manual of Mental Disorders; MCMI II: Millon Clinical Multiaxial Inventory; NPD: Narcissistic Personality Disorder; PD: Personality Disorder; SPSS: Statistical Package for the Social Sciences.

## Competing interests

The authors declare that they have no competing interests.

## Authors' contributions

CSB conceived the study and participated in its design. JMTM contributed to the acquisition of data and to the drafting and revision of the manuscript. MOLL participated in the analysis and interpretation of data. All authors read and approved the final manuscript.

## Pre-publication history

The pre-publication history for this paper can be accessed here:

http://www.biomedcentral.com/1471-244X/11/192/prepub
